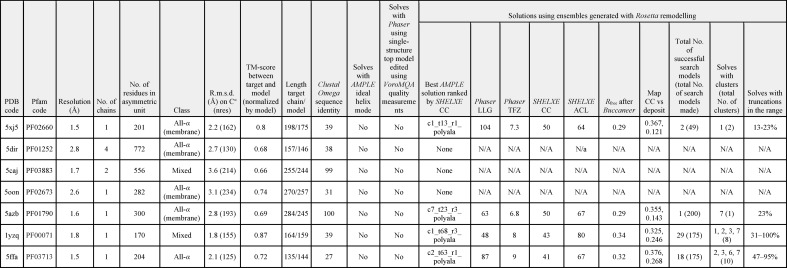# Molecular replacement using structure predictions from databases

**DOI:** 10.1107/S2059798319013962

**Published:** 2019-11-19

**Authors:** Adam J. Simpkin, Jens M. H. Thomas, Felix Simkovic, Ronan M. Keegan, Daniel J. Rigden

**Affiliations:** a Institute of Integrative Biology, University of Liverpool, Liverpool L69 7ZB, England; b STFC, Rutherford Appleton Laboratory, Research Complex at Harwell, Didcot OX11 0FA, England

**Keywords:** *ab initio* modelling, databases, molecular replacement, *ab initio* structure predictions

## Abstract

Molecular replacement (MR) is the predominant route to solution of the phase problem in macromolecular crystallography. Where the lack of a suitable homologue precludes conventional MR, one option is to predict the target structure using bioinformatics. Such modelling, in the absence of homologous templates, is called *ab initio* or *de novo* modelling. Recently, the accuracy of such models has improved significantly as a result of the availability, in many cases, of residue-contact predictions derived from evolutionary covariance analysis. Covariance-assisted *ab initio* models representing structurally uncharacterized Pfam families are now available on a large scale in databases, potentially representing a valuable and easily accessible supplement to the PDB as a source of search models. Here, the unconventional MR pipeline *AMPLE* is employed to explore the value of structure predictions in the GREMLIN and PconsFam databases. It was tested whether these deposited predictions, processed in various ways, could solve the structures of PDB entries that were subsequently deposited. The results were encouraging: nine of 27 GREMLIN cases were solved, covering target lengths of 109–355 residues and a resolution range of 1.4–2.9 Å, and with target–model shared sequence identity as low as 20%. The cluster-and-truncate approach in *AMPLE* proved to be essential for most successes. For the overall lower quality structure predictions in the PconsFam database, remodelling with *Rosetta* within the *AMPLE* pipeline proved to be the best approach, generating ensemble search models from single-structure deposits. Finally, it is shown that the *AMPLE*-obtained search models deriving from GREMLIN deposits are of sufficiently high quality to be selected by the sequence-independent MR pipeline *SIMBAD*. Overall, the results help to point the way towards the optimal use of the expanding databases of *ab initio* structure predictions.

## Abstract 2

Predicted *ab initio* protein models from online databases are a useful supplement to the PDB for molecular replacement, but usually require nontrivial processing to succeed.

## 1. Introduction   

Macromolecular crystallography requires a source of phasing information to supplement the measured diffraction intensities and thereby solve a structure. Although experimental methods are available, the most popular method for obtaining phase information is molecular replacement (MR). MR involves the positioning of a search model in the asymmetric unit, usually by sequential rotation and translation steps, thereby providing approximate phase information which, together with the measured diffraction data, allows the calculation of initial electron-density maps (Rossmann & Blow, 1962 ▸).

Conventional MR typically employs the structure of a homologue of the target protein as a search model, often after some manual or automatic editing. The editing is designed to remove loops or side chains that are shown by sequence comparison to differ between homologue and target, or which are flexible and hence prone to adopt different conformations in the known and unknown structures (Schwarzenbacher *et al.*, 2004 ▸; Stein, 2008 ▸; Bunkóczi & Read, 2011 ▸; Lebedev *et al.*, 2008 ▸). Conventional MR becomes more difficult as the target–search model relationship becomes more distant and, consequently, the structures tend to differ more. Considerable effort has therefore been applied to push the boundaries of conventional MR by nontrivial treatments of distantly homologous structures (Bunkóczi & Read, 2011 ▸; Rigden *et al.*, 2018 ▸; Sammito *et al.*, 2014 ▸) and/or their advantageous superposition to serve as ensemble search models (Leahy *et al.*, 1992 ▸; Adams *et al.*, 2010 ▸; Keegan *et al.*, 2018 ▸). Ensemble search models work particularly effectively with the maximum-likelihood scoring approach used by *Phaser* (McCoy, 2004 ▸; McCoy *et al.*, 2007 ▸). The selection of homologues to serve as search models is typically performed by a sequence-homology search of the Protein Data Bank (PDB; wwPDB Consortium, 2018 ▸), but the imperfect correlation between sequence and structural similarity (for example in protein families that can adopt multiple conformations) means that large-scale sequence-independent screens of the PDB or a derivative database are also undertaken (Hatti *et al.*, 2016 ▸; Stokes-Rees & Sliz, 2010 ▸; Simpkin *et al.*, 2018 ▸).

Beyond the boundaries of conventional MR, for very distant homologues or even novel folds, unconventional MR approaches have been developed. These exploit other sources of search models such as ideal regular secondary-structure elements or motifs (Rodríguez *et al.*, 2012 ▸), recurring tertiary folding patterns (Sammito *et al.*, 2013 ▸) or *ab initio* models (Bibby *et al.*, 2012 ▸; Keegan *et al.*, 2015 ▸; Simkovic *et al.*, 2016 ▸). *Ab initio* models are structure predictions that can be obtained based on sequence alone, independent of structural information from homologues present in the PDB. The first broadly successful approach, as used by the programs *Rosetta* (Shortle *et al.*, 1998 ▸; Leaver-Fay *et al.*, 2011 ▸) and *QUARK* (Xu & Zhang, 2012 ▸), builds structures from fragments of unrelated proteins using Monte Carlo algorithms to sample search space and sophisticated search functions to recognize structures that share features of experimental protein structures. Early work on the use of *ab initio* models (Qian *et al.*, 2007 ▸; Rigden *et al.*, 2008 ▸) inspired the development of the *AMPLE* pipeline using *Rosetta* in particular for the modelling (Bibby *et al.*, 2012 ▸). However, its utility was limited by the size of protein that could be accurately modelled, up to around 120 residues at the time, and by the poorer quality in general of models that were rich in β-structure, in comparison to α-helical proteins (Bibby *et al.*, 2012 ▸).

More recently, the availability of intramolecular and intermolecular residue-contact predictions, derived from evolutionary covariance analysis of deep protein-sequence alignments (Morcos *et al.*, 2011 ▸), has revolutionized structural bioinformatics (de Oliveira & Deane, 2017 ▸), with many implications for structural biology (Simkovic *et al.*, 2017 ▸). It was immediately perceived that good-quality contact predictions would enable the folding *ab initio* of much larger proteins (Marks *et al.*, 2011 ▸). Indeed, reasonably accurate fold predictions were soon obtained for globular proteins of >200 residues (Marks *et al.*, 2011 ▸) and transmembrane helical proteins containing more than 500 residues (Hopf *et al.*, 2012 ▸). Several groups use the distance geometry structure-prediction methods implemented in *CNS* (Brünger *et al.*, 1998 ▸; Brunger, 2007 ▸), but others have continued with fragment-assembly approaches, with particularly impressive results obtained by exploiting metagenomics databases to deepen the sequence alignments that can be obtained for targets and thereby obtain more accurate contact predictions (Ovchinnikov *et al.*, 2017 ▸).

**Figure 1:**
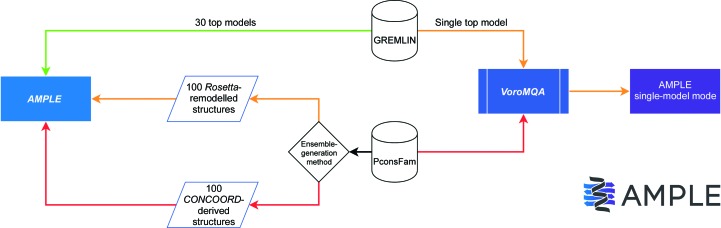
Flowchart showing the methods used to treat search models obtained from GREMLIN and PconsFam prior to *AMPLE* or *AMPLE* single-model mode. The relative success of each method is represented in green, orange or red, where green represents a more successful method and red represents a less successful method.

With the rapid development of contact-assisted *ab initio* modelling methods, several groups have given thought to producing structure predictions to cover protein sequence space, using Pfam (El-Gebali *et al.*, 2018 ▸) as a convenient definition of protein families. Prominent among these are the GREMLIN database (Ovchinnikov *et al.*, 2017 ▸), which contains representatives of 614 Pfam families resulting from sophisticated iterative modelling with *Rosetta*, and the PconsFam database (Lamb *et al.*, 2019 ▸), which covers a much larger number of protein families, 13 617, but with more rapidly obtained models. Since these models represent Pfam families, often with thousands of members, they provide a degree of structural information for many proteins: for example, the GREMLIN authors calculate that their models with predicted TM-scores of >0.65 (where a TM-score of >0.5 is taken as a correct fold prediction; Zhang & Skolnick, 2004*a* ▸; Xu & Zhang, 2010 ▸) cover almost half a million sequences in UniRef100 (Suzek *et al.*, 2007 ▸). Thus, as models have become increasingly accurate, and especially as they are likely to become ever more readily accessible in prominent protein-sequence databases in the near future, an exploration of their potential for MR is timely. Here, we show that the *AMPLE* MR pipeline provides an effective way to prepare search models from entries in the GREMLIN and PconsFam databases. The former are clustered and truncated directly using the same protocols as developed for locally produced *ab initio* models: this solves many more structures than using deposited structure predictions more directly. The single deposited structure predictions in the PconsFam database are best dealt with by *Rosetta* remodelling, which can be conveniently performed within the *AMPLE* pipeline, with clustering and truncating of the results to compose ensemble search models (Fig. 1 ▸). A preliminary exploration of the use of database-derived search ensembles in the sequence-independent MR pipeline *SIMBAD* (Simpkin *et al.*, 2018 ▸) is also presented.

## 2. Methods   

### 2.1. Test-set selection   

Cases were chosen from the GREMLIN database (Ovchinnikov *et al.*, 2017 ▸), which contains 30 structure predictions for each of 614 proteins, each of which represents a Pfam family (El-Gebali *et al.*, 2018 ▸) that was structurally uncharacterized (*i.e.* the Pfam database recorded no experimentally determined structure in the family entry) at the time of modelling. At the time of publication of the database, structures had subsequently been determined for six families. 30 families that were structurally characterized post-modelling between January 2017 and December 2018 were identified by mining the Pfam database for structures related to the 614 families. This gave a total of 36 (Supplementary Table S1). Of these, nine were eliminated as only having diffraction data to >3 A resolution (one case) or where the quality of the model was too poor (nine cases). Poor modelling was defined as resulting in models (represented by the first of the 30 structures deposited for each protein) that gave TM-scores (Zhang & Skolnick, 2004*a* ▸), normalized either to the target structure or to the model, that were both <0.5: such values indicate that the overall fold has not been correctly modelled (Xu & Zhang, 2010 ▸). We asked whether the remaining 27 cases (Table 1 ▸) could have been solved using the results of the modelling deposited in the databases.

The PconsFam database (Lamb *et al.*, 2019 ▸) contains single-structure predictions for 13 617 proteins, again each representing a Pfam family. As well as addressing novel folds, it contains models for families that have been structurally characterized. For 22 of the 27 cases above models were available from the PconsFam database. However, only six of the 22 passed the TM-score of >0.5 criterion and one of these (PDB entry 4xb6) was not attempted since the models were rather poor (TM-score of 0.55) and eight copies of the target protein were present in the asymmetric unit. Since the number of suitable PconsFam models was rather small, experiments were also undertaken with selected other families for which it was known that high-quality models were available in the PconsFam database. These were the Ras family (PF00071), where the model was used to try to solve the structure deposited in the PDB as entry 1yzq (1.78 Å resolution), and the DUF305 family (PF03713; PDB entry 5ffa; 1.50 Å resolution).

### 2.2. Search-model generation   

For the 27 GREMLIN test cases, the 30 structure predictions deposited for each were used as direct input to *AMPLE* v.1.4.6 in *CCP*4 v.7.0.68 (Winn *et al.*, 2011 ▸). The current default processing options were used for search-model composition: namely, for each of the top ten *SPICKER* (Zhang & Skolnick, 2004*b* ▸) clusters, truncate progressively in 20 steps from 100% (untruncated) down to around 5% remaining, subcluster (Bibby *et al.*, 2012 ▸) using 1 or 3 Å radii and remove all side chains to leave polyalanine search models. Models are truncated into bins as close to the desired percentage intervals as possible, but as protein sequences are discrete entities of variable lengths they are not always evenly divisible into the desired bins. As the actual size of the truncation bins is reported, the size of the bins may vary a little from the ideal percentage values.

Two additional attempts were made for comparison: all 30 structure predictions were presented directly to *Phaser* as an ensemble, and entries in the separate database of single ‘final models’ were processed in *AMPLE* single-structure mode (Rigden *et al.*, 2018 ▸) using *VoroMQA* (Olechnovič & Venclovas, 2017 ▸) to provide per-residue quality scores which drove progressive truncation over a set of 20 thresholds. Retention of side chains or editing to polyalanine were specified so that 40 search models were derived for each case.

Since the PconsFam database contains only single models per Pfam family, three approaches were tried. Firstly, truncation of the single models in *AMPLE* was performed using its single-structure mode as above using *VoroMQA* protein structure-quality predictions. Secondly, *Rosetta* remodelling was performed using the PconsFam model as a basis. This approach was previously employed with NMR ensembles and proved to improve performance. Using the -nmr_remodel flag causes *AMPLE* to idealize the input structure, here the PconsFam model, and then remodel the result, using a provided target sequence, into a number of new structures, sampling conformational space in a fragment-dependent fashion. Fragment libraries were obtained from the *Robetta* server (Kim *et al.*, 2004 ▸) with the ‘exclude homologues’ option selected in order that the remodelling was not influenced by any knowledge of the target structure or homologues. Here, 100 structures were derived from each PconsFam model and given to *AMPLE* for clustering and truncation as above. Thirdly, for selected targets, the PconsFam single structures were transformed into ensembles using *CONCOORD* (de Groot *et al.*, 1997 ▸) as described previously (Rigden *et al.*, 2018 ▸). Briefly, *CONCOORD* extracts restraints from a given structure and then uses distance-geometry methods to build a set of variant structures that differ from the original but which obey the derived restraints. Using this procedure, less well packed regions such as loops exhibit structural divergence in the resulting derivative structures and hence, by the *AMPLE* algorithm, are subject to truncation.

### 2.3. Molecular replacement   

Within the *AMPLE* pipeline, *MrBUMP* (Keegan *et al.*, 2018 ▸) trialled the search models using *Phaser* v.2.8.2 (McCoy *et al.*, 2007 ▸; Read & McCoy, 2016 ▸). The default *AMPLE*-estimated r.m.s.d. error of 0.1 Å was used, but this value was adjusted internally by *Phaser* where inconsistent with the internal structural variability of the ensemble. Success was judged as a placement that yielded a map correlation coefficient (CC) of 0.25 or higher using *phenix.get_map_cc_mtz_pdb* (Adams *et al.*, 2010 ▸). All of these cases also produced a CC of >25% upon main-chain tracing using *SHELXE* (Thorn & Sheldrick, 2013 ▸), with the single exception of PDB entry 5uw2, for which diffraction data to only 2.9 Å resolution were available, which produced a marginally lower score of 24.8. All of these solutions could be refined to an *R*_free_ of <0.45 using either just the *Buccaneer* (Cowtan, 2006 ▸) plus *REFMAC* (Murshudov *et al.*, 2011 ▸) protocol built into the default operation of *AMPLE* or, where necessary (for PDB entries 5oon and 5uw2), by directly refining the *Phaser* placement with *REFMAC* (Murshudov *et al.*, 2011 ▸), or with manual model building. For comparison, we attempted solution of all 27 using the ideal-helix mode of *AMPLE* with a *Phaser* time limit per search model of 24 h.

### 2.4. *SIMBAD*   

*SIMBAD* is an MR pipeline that uses the rotation function to screen large databases of structures (Simpkin *et al.*, 2018 ▸). *SIMBAD* has recently been modified to run the likelihood-enhanced fast rotation function in *Phaser* (Simpkin *et al.*, 2019 ▸). This increased the sensitivity of the pipeline and also allowed single search models to be replaced with ensembles. The *MoRDa* (Vagin & Lebedev, 2015 ▸) ensemble database that *SIMBAD* is typically run against was modified to include *AMPLE*-derived ensembles made from the models in the GREMLIN database. Initial experiments suggested that the rotation function was not sensitive enough to pick up these poor models, so *SIMBAD* was modified to also run the likelihood-enhanced fast translation search in *Phaser* (McCoy *et al.*, 2005 ▸) but only on the best orientation identified in the rotation function. In this work, the top 200 solutions by translation score were taken forward for MR and refinement, as opposed to the top 200 solutions by rotation score in previously published work.

## 3. Results and discussion   

### 3.1. Using models from the GREMLIN database   

The 27 cases studied include many cases that are challenging in terms of the relatively high structural deviations between model and target and/or the complex and sometimes heterooligomeric composition of the asymmetric unit: only eight cases contained a single chain in the asymmetric unit. When the GREMLIN structure predictions, each comprising 30 models of a given protein representing a particular Pfam family, were supplied to *AMPLE* for its default clustering and truncation approach, nine of the 27 cases were solved (Supplementary Table S1). These nine cases include four transmembrane helical proteins, one globular helical protein and four mixed-fold proteins. Thus successes spanned all fold classes, but the numbers are too small to suggest whether certain types of protein may be particularly (un)favourable. The ultimately successful structure predictions overall can be considered of medium quality, sharing an r.m.s.d. of 1.5–2.8 Å on C^α^ atoms (TM-scores of 0.63–0.84) with the targets. The solved cases cover a range of lengths of 112–355 residues and a resolution range of 1.35–2.85 Å.

In most cases, the modelled member of a given Pfam family was closely related (>90% shared sequence identity) to the member ultimately structurally characterized. However, there were three exceptions. The first was PDB entry 5cuo, the crystal structure of *Rhodopseudomonas palustris* PduL, which was solved with models of phosphate propanoyltransferase from *Bacillus megaterium* (Pfam PF06130, UniProt D5DKA5), with which it shared only 49% sequence identity. The second was PDB entry 5xj5, the structure of *Aquifex aeolicus* glycerol-3-phosphate acyltransferase, where the model from *B. subtilis* (Pfam PF02660, UniProt Q45064) shared only 36% sequence identity with the target. Most remarkable was PDB entry 5mlz, the structure of dolichyl-phosphate mannose synthase, where the model of an uncharacterized GtrA-family protein from *B. subtilis* (Pfam PF04138, UniProt O31821) shared only 20% sequence identity with the target. When considering these successes with relatively distant homologues, it is worth remembering that the covariance signal, which strongly influences the modelling, will be strongest for features that are shared throughout the superfamily. This may well help to produce models that serve to solve targets from across a superfamily. However, it is also true that the GREMLIN structure predictions are derived from an all-atom, fully sequence-aware protocol that would be expected to give authentically different predictions for homologous proteins. As such, it remains encouraging that structure predictions can solve quite distantly homologous targets. In the three cases mentioned here the secondary structure of the GREMLIN prediction matched that of the target quite well (Supplementary Figs. S1–S3).

As expected, cases with multiple chains in the asymmetric unit solved less often, but *AMPLE* succeeded with PDB entry 5caj (two chains) and PDB entry 5uw2 (three chains). Since some of the targets contained multiple domains, the search models sometimes represented only a portion of the target. Such was the case with PDB entry 5mlz, where the available model was 123 residues long but solved a structure of 352 residues.

The ease of solution of the nine cases, expressed as the proportion of search-model ensembles that succeeded, varies widely. For PDB entry 5edl 132 out of 170 search models (78%) succeeded, while for PDB entry 5caj the success rate was six out of 132 (4.5%). PDB entry 5edl solved with search models containing 11–100% of the starting-model residues, while others solved over a narrower range of search-model sizes: 27–41% for PDB entry 5mlz, for example. The most truncated successful search model contained 7% of the starting structure (19 residues) of the model (PDB entry 5azb). This target is the structure of *E. coli* lipoprotein diacylglyceryl transferase, an integral membrane enzyme of 300 residues in length, determined to a resolution of 1.6 Å. The 7% successful search model comprised an antiparallel pair of helices. Successful search models for a given target tended to derive from different clusters, but cluster 1, containing the largest number of the input 30 models, was not always successful: PDB entry 5cuo, for example, only solved with search models deriving from clusters 2 and 3. Overall, the results suggest that the cluster-and-truncate approach in *AMPLE*, intensively sampling many nontrivial edits of ensembles deriving from the deposited models, is an appropriate strategy to deal with these structures.

The need to use the automated processing and sampling in *AMPLE* for the best performance is illustrated by the poorer performance of two simple baseline approaches. When the top model for each protein, provided separately to the ensembles in the GREMLIN database, was used, using *VoroMQA* quality measurements to produce a series of truncated derivatives, only two cases were solved, PDB entries 5mlz and 5edl. Secondly, when the 30 structures were presented as an ensemble to *Phaser* directly, only one case was solved. The successful case was PDB entry 5edl, where the models in the ensemble had an r.m.s.d. of between 1.59 and 2.30 Å (TM-scores of 0.4–0.87) from the true structure.

**Figure 2:**
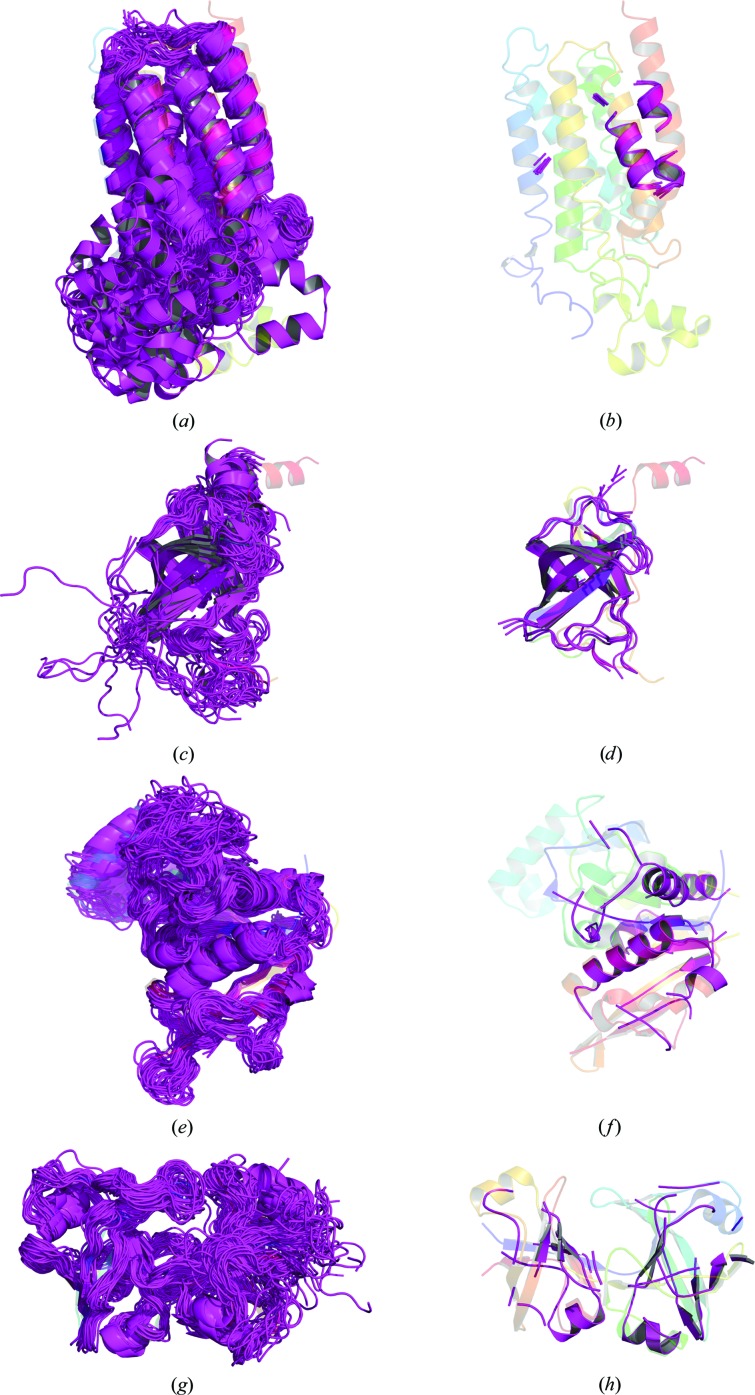
(*a*) The 30 models obtained from the GREMLIN database for PF01790 (magenta) aligned with the crystallized structure, PDB entry 5azb (rainbow from blue at the N-­terminus to red at the C-terminus). (*b*) The best-performing *AMPLE*-derived ensemble (magenta), derived by truncating cluster 1 down to 12% (33 residues), aligned with the crystallized structure, PDB entry 5azb (rainbow). (*c*) The 30 models obtained from the GREMLIN database for PF02470 (magenta) aligned with the crystallized structure, PDB entry 5uw2 (rainbow). (*d*) The best-performing *AMPLE*-derived ensemble (magenta), derived by truncating cluster 2 down to 80% (96 residues), aligned with the crystal structure, PDB entry 5uw2 (rainbow). (*e*) The 30 models obtained from the GREMLIN database for PF03883 (magenta) aligned with the crystallized structure, PDB entry 5caj (rainbow). (*f*) The best-performing *AMPLE*-derived ensemble (magenta), derived by truncating cluster 1 down to 54% (137 residues), aligned with the crystallized structure, PDB entry 5caj (rainbow). (*g*) The 30 models obtained from the GREMLIN database for PF06130 (magenta) aligned with the crystallized structure, PDB entry 5cuo (rainbow). (*h*) The best-performing *AMPLE*-derived ensemble (magenta), derived by truncating cluster 3 down to 70% (138 residues), aligned with the crystallized structure, PDB entry 5cuo (rainbow).

The successes presented undoubtedly cover targets that could potentially have been solved alternatively using fragment-based approaches (Rodríguez *et al.*, 2009 ▸; Jenkins, 2018 ▸). Although the simple ideal-helix mode in *AMPLE* performed relatively poorly, only solving three targets, more sophisticated approaches might well perform better, particularly for cases with higher resolution diffraction data, helix-rich composition and/or small asymmetric unit contents. The more challenging cases to be solved therefore include PDB entry 5cuo, a largely β-structure containing two ∼200-residue chains, PDB entry 5uw2, with diffraction only to 2.9 Å resolution, and PDB entry 5caj, where diffraction data to 1.65 Å resolution were available but the asymmetric unit contained 510 residues. Fig. 2 ▸ illustrates that the most successful search models in these three cases are only moderately truncated down to 54%, 70% or 80% of the starting structures, indicating that correct overall fold prediction is important (see also Supplementary Figs. S4–S6). In contrast, the best-performing search model for PDB entry 5azb (Fig. 2 ▸) contained only 12% of the starting structure, and truncations to below 33% were required for success (Supplementary Fig. S7). This observation demonstrates the importance of the sampling by *AMPLE* of truncations over a wide range.

### 3.2. Using models from the PconsFam database   

Applying the same TM-score threshold of 0.5, indicating a broadly correct predicted fold (Xu & Zhang, 2010 ▸), only five of the 27 families considered above were represented by PconsFam structure predictions that were good enough to take to MR trials. PconsFam contains only single structure predictions for representative proteins of Pfam domains. Three different strategies were therefore employed: truncation of that single structure according to local model-quality prediction from the *VoroMQA* server, generation of ensembles using the distance-geometry method *CONCOORD* and *Rosetta* remodelling using the PconsFam deposition as a starting point.

**Figure 3:**
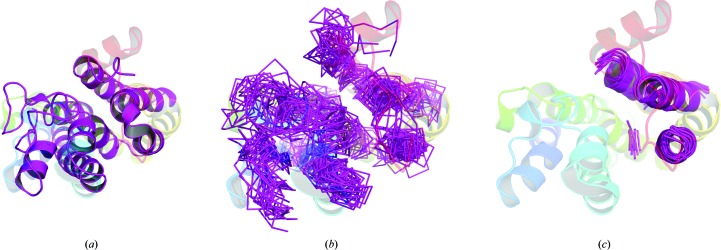
(*a*) PconsFam model for PF02660 (magenta) aligned with the crystallized structure, PDB entry 5jx5 (rainbow). (*b*) An untruncated *AMPLE* ensemble (magenta ribbon), following *Rosetta* remodelling, aligned with the crystallized structure, PDB entry 5jx5 (rainbow). (*c*) The truncated *AMPLE* ensemble (c1_23_r3_polyAla) obtained from the *Rosetta*-remodelled versions of the PconsFam model for PF02660 (magenta) aligned with the crystallized structure, PDB entry 5jx5 (rainbow).

The simplest approach, editing a single model according to per-residue predicted quality scores, failed to solve any of the five targets. *Rosetta* remodelling was successful with two of the five, PDB entries 5xj5 and 5azb, both of which are transmembrane helical proteins. PDB entry 5xj5 solved with two search models out of 49, which were truncated ensembles from the first cluster containing 23 or 41 residues. The *SHELXE* traces were automatically rebuilt using *Buccaneer* within the *AMPLE* pipeline to final *R*_free_ values of 28–29%. The larger search model, c1_23_r3_polyAla (where c1 means derived from cluster 1, 23 means that 23% of the initial model remains, r3 refers to a 3 Å subclustering radius and polyAla refers to the side-chain treatment), contains most of the C-terminal three-helical subdomain of the target structure, which is more accurately predicted (Fig. 3 ▸). PDB entry 5azb was solved by a single search model from the 200 produced. It was derived from the seventh cluster and truncated until it contained 57 residues, which mainly composed portions of four of the transmembrane helices. Again, automated rebuilding produced an *R*_free_ of 29%. Neither of these cases was solved by the simpler and somewhat less time-consuming approach of ensemble generation with *CONCOORD*.

In order to further explore approaches that could convert PconsFam models into successful search models, some trials were performed with Ras protein (Pfam accession PF00071; PDB entry 1yzq) and DUF305 (PF03713; PDB entry 5ffa). For these, high-quality structure predictions were available with TM-scores of 0.85 and 0.76, respectively, and both solved using *Rosetta* remodelling. The Ras structure was solved with 29 of 175 search-model ensembles generated, deriving from clusters 1, 2, 3 or 7, containing 53–170 residues (170 residues being the full size of the model) and tracing and refining to *R*_free_ values as low as 33% within the *AMPLE* pipeline. The DUF305 structure solved with 18 of 175 search-model ensembles. These were derived from clusters 2, 3, 6 or 7, contained between 79 and 143 residues and automatically traced and refined to *R*_free_ values as low as 33% (Table 2). ▸

**Figure 4:**
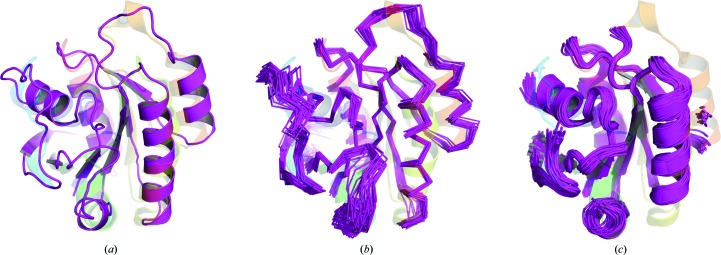
(*a*) PconsFam model for PF00071 (magenta) aligned with the crystallized structure, PDB entry 1yzq (rainbow). (*b*) An untruncated *AMPLE* ensemble (magenta ribbon), following *CONCOORD*, aligned with the crystallized structure, PDB entry 1yzq (rainbow). (*c*) The *AMPLE* ensemble obtained from the *CONCOORD* derivatives for PF00071 (magenta) aligned with the crystallized structure, PDB entry 1yzq (rainbow).

Interestingly, *CONCOORD*-derived ensembles could solve the Ras structure but not the DUF305 case. In the successful run, seven search-model ensembles out of a total of 400 generated were successful, deriving from clusters 5, 7, 8 or 9 and containing 50–75% of the original model, corresponding to 79–119 residues. Although deriving from different clusters, the successful search models were similar in having discarded less accurately modelled loops but retaining the core fold of well captured secondary-structure elements (Fig. 4 ▸).

Several factors could be contributing to the relative success of the *Rosetta* remodelling approach compared with the single PconsFam model. Most obviously, remodelling the target sequence could take the structure closer to that of the target, especially in cases where the sequence identity between the target and the PconsFam deposition is low. This would combine with the use of a sophisticated energy function in *Rosetta* (Alford *et al.*, 2017 ▸), rather than the simpler function used by *CONFOLD*, the structure-building algorithm in PconsFam (Adhikari *et al.*, 2015 ▸), to potentially allow more accurate modelling, *i.e.* the PconsFam structure might be ‘refined’ by the *Rosetta* step. Secondly, modelling based on covariance information-guided distance-geometry methods, as in PconsFam, can often lead to results in which local backbone geometry is poor. Potentially, the backbone geometry could be improved by running through the fragment-based remodelling in *Rosetta*. Finally, as has been well established (Qian *et al.*, 2007 ▸; Rigden *et al.*, 2008 ▸), comparison across the multiple structures resulting from remodelling allows the inference of quality, enabling truncation to more accurately modelled core regions. Supplementary Table S3 shows the overall accuracy and stereochemical quality of the PconsFam models and the *Rosetta* structures derived from them.

The results confirm a clear and consistent improvement in backbone geometry as measured by Ramachandran plot statistics and an overall *G*-factor calculated on backbone dihedrals, with positive values indicating better quality. However, these suggest that *Rosetta* does not generally act to refine the PconsFam models: in fact, in three of the four cases the average correctness of the models, measured as TM-scores, is worse than for the PconsFam starting model. Where the starting structure is poorer quality, it seems that *Rosetta* fragment-based conformational exploration can effectively unfold the structure. Options to try to prevent this in the future could include the imposition of evolutionary covariance-derived contact predictions or more generalized restraints to maintain the structure in the vicinity of the starting model. Nevertheless, the *AMPLE* protocol, being based on clustering, is tolerant of some unfolded structures among the input set.

Overall, the results suggest that simple editing of the single-structure PconsFam models is unlikely to transform them into successful search models. However, where the overall fold has been correctly captured, *Rosetta* remodelling with subsequent clustering and truncating to generate ensembles can be effective. This approach clearly outperforms *CONCOORD* for ensemble generation.

### 3.3. *SIMBAD* and search models derived from databases   

*SIMBAD* is a sequence-independent MR pipeline that attempts to solve structures using a lattice search, a search of a curated database of known contaminant structures and/or a large-scale search of domain structures (around 120 000) from the *MoRDa* database. Since recent developments in *SIMBAD* (Simpkin *et al.*, 2019 ▸) have improved its sensitivity, by using *Phaser* in place of the original *AMoRe* and through the use of ensemble search models, we tested whether truncated search-model ensembles derived from the GREMLIN database that succeeded in *AMPLE* could also succeed in *SIMBAD*.

Success in the large-scale *MoRDa* screen can arise in two ways in *SIMBAD*. Firstly, if a tested search model yields a *Phaser* RFZ that is high enough (>7) to generally indicate an accurate rotation then it is immediately trialled in a full MR protocol, the success of which (*R* values below 0.45 and/or both LLG > 120 and TFZ > 8) would lead to the termination of *SIMBAD* without testing any remaining search models. Alternatively, if no search model reaches the RFZ threshold, then at the end of the rotation-function screen of all search models the 200 that have the highest RFZ scores are trialled for full MR.

The GREMLIN structure predictions are of moderate accuracy at best and require significant processing to succeed. Therefore, we first assessed whether they would score RFZ values likely to lead to their selection in the top 200 in a full *MoRDa* + GREMLIN run. Supplementary Table S4 shows the range of RFZ values obtained for the range of truncated search models produced by *AMPLE* for cases that successfully solved. In general the results were somewhat disappointing: no search-model ensemble achieved an RFZ of greater than 6.11. Although full *SIMBAD* runs were not performed, experience suggests that these values are unlikely to place the search-model ensembles, even those that ultimately succeeded in *AMPLE*, within the top 200. As such, they would never proceed to the full MR step.

In a bid to improve the sensitivity of the *SIMBAD* pipeline further, we therefore experimented with the addition of the *Phaser* translation function on just the top-ranked orientation in the rotation search. We reasoned that placing the search model would improve the signal to noise from good search models. Preliminary results suggested that this worked well: for example, search-model ensembles for PDB entry 5xj5 gave LLG and TFZ scores of up to 90.35 and 7.68, respectively, while the ensembles for PDB entry 5edl gave LLG and TFZ scores of as high as 147.32 and 13.05, resepectively. These values are indicative of success.

**Figure 5:**
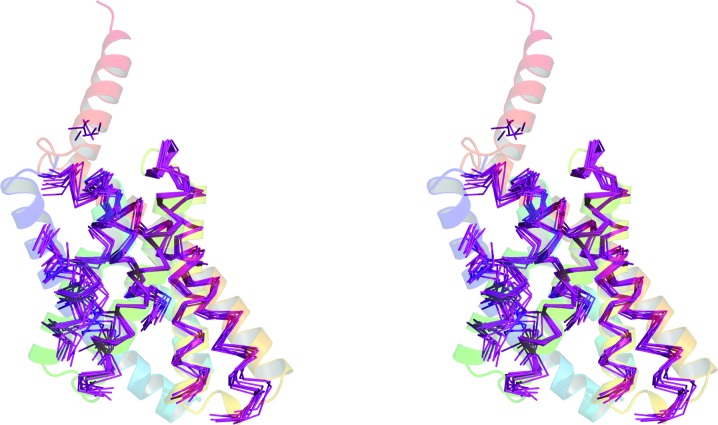
Cross-eyed stereoview of the *AMPLE* ensemble (c1_t74_r3_polyAla) which gave the best score in the *SIMBAD* search for PF09819 (magenta) aligned with the crystallized structure, PDB entry 5edl (rainbow from blue at the N-terminus to red at the C-terminus).

A version of *SIMBAD* in which the database, in this case *MoRDa* supplemented by GREMLIN-derived ensembles, is screened using a rotation function in combination with the rapid translation function was then produced. As a proof of principle this was tested on PDB entry 5edl owing to the high TFZ scores that were observed. This gave a clear success, with six *AMPLE* ensembles being reported in the top 200 (c1_74_r3_polyAla, c1_t89_r3_polyAla, c1_t74_r1_polyAla, c1_t79_r1_polyAla, c1_t84_r3_polyAla and c1_t100_r3_polyAla), with the best example being shown in Fig. 5 ▸.

Naturally, the additional translation function can increase the runtime of *SIMBAD*, but this will be compensated for, to some extent, by more frequent early termination owing to the improved sensitivity with which good search models can be selected.

## 4. Conclusions   

Databases of protein homology models have a long history (Kiefer *et al.*, 2009 ▸; Pieper *et al.*, 2014 ▸; Guex & Peitsch, 1997 ▸), most recently under the aegis of the Protein Model Portal (Haas *et al.*, 2013 ▸), and homology models have been used for MR (see, for example, Horsefield *et al.*, 2008 ▸; Jung *et al.*, 2011 ▸). Nevertheless, we are unaware of cases in which a homology model, much less an *ab initio* model, downloaded from a database has been used as a search model. These new results demonstrate that the recently emerged databases of *ab initio* models, representing Pfam families with structures that are very different from anything deposited in the PDB, already contain information that can solve the structures of proteins from these families by MR. The success of the MR in *AMPLE* should be considered in the context of the quality of the models available in the GREMLIN and PconsFam databases. We could collect 36 cases representing Pfam families that were not structurally characterized at the time of their GREMLIN modelling but were subsequently deposited in the PDB. Of these, 27 had GREMLIN models with the correct fold (TM-score of >0.5), while the figure was only five for the PconsFam database. This observation can be related to the more sophisticated modelling protocol behind the GREMLIN database and its exploitation of metagenomic data to improve the quality of the contact predictions driving the modelling (Ovchinnikov *et al.*, 2017 ▸). However, within those different sets the success by MR was actually comparable: nine out of 27 with GREMLIN-derived search models and two out of five with PconsFam. GREMLIN predictions with TM-scores as low as 0.64 could succeed, while the two successful PconsFam cases in the set of five were based on structure predictions with TM-scores of 0.80 and 0.69; for the additional PconsFam cases (Ras and DUF305) these values were 0.85 and 0.76. Overall, the results suggest that models should score somewhat better than the correct fold criterion of a TM-score of >0.5 in order to succeed. The current advantage of the PconsFam databases is its coverage, but the simpler modelling protocol is likely to mean that its predictions are of poorer quality on average than the GREMLIN contents. A user may currently estimate the likely model quality of a PconsFam model by looking at its Pcons (Lundström *et al.*, 2001 ▸) or ProQ3D (Uziela *et al.*, 2017 ▸) model-quality scores, or the underlying alignment depth (number of effective sequences) upon which the contact prediction was performed.

The requirement of the *Rosetta* remodelling approach for success with some PconsFam models might invite the comment that a user could simply generate their own models rather than work with those from the database. However, databases such as PconsFam and GREMLIN contain models derived using state-of-the-art contact predictions and, in the latter case, complex, bespoke and iterative modelling pipelines. For a crystallographer to recapitulate these approaches, within or without *AMPLE*, is certainly more demanding in computational skills and infrastructure than the comparatively rapid (around 80 min on ten cores) remodelling approach that is outlined here.

In summary, these results demonstrate that *ab initio* structure predictions deposited in online databases are already of sufficient quality to form the basis of successful MR search models. Some of the targets addressed here could undoubtedly be alternatively solved using sophisticated fragment-based methods (Rodríguez *et al.*, 2009 ▸; Jenkins, 2018 ▸), but *AMPLE* conveniently provides a unifying framework to attempt the solution of such cases (typically higher resolution and higher helical content, *e.g.* Fig. 2*a* ▸), as well as harder cases (Figs. 2*b*–2*d* ▸) where high-quality modelling is key and moderately edited search models containing almost entire folds succeed. However, the evidence currently suggests that nontrivial processing is required for optimal performance to transform single models into ensembles and to eliminate inaccurate regions from ensembles such that better modelled core regions remain. These *ab initio* models are calculated using covariance-driven approaches and represent sometimes large families of structurally uncharacterized proteins. The GREMLIN database has much smaller coverage at the time of writing, but there are plans to liaise with the Pfam database (El-Gebali *et al.*, 2018 ▸) and use the latter as a means to disseminate models that cover more of protein-sequence space. Such models will be periodically recalculated as and when the expansion of sequence databases allows improved contact predictions and hence better modelling (R. Finn, personal communication). These plans run alongside similar efforts to collect homology models from structural bioinformatics resources such as Genome3D (Lewis *et al.*, 2013 ▸) and make them available within the InterPro database (Mitchell *et al.*, 2019 ▸; R. Finn, personal communication). In the near future these databases will facilitate access to increasingly available and high-quality models, be they *ab initio*-based or homology-based. As such, they will increasingly be viewed as a valuable supplement to the PDB as sources of MR search models.

## 5. Related literature   

The following references are cited in the supporting information for this article: Kabsch & Sander (1983 ▸), Thompson *et al.* (2002 ▸), Waterhouse *et al.* (2009 ▸) and Zhang & Skolnick (2005 ▸).

## Supplementary Material

Supplementary Figures and Tables. DOI: 10.1107/S2059798319013962/rr5187sup1.pdf

Supplementary Tables S1 and S4. DOI: 10.1107/S2059798319013962/rr5187sup2.xlsx

## Figures and Tables

**Table 1:** Results for the 27 test cases that were trialled in *AMPLE* using GREMLIN models

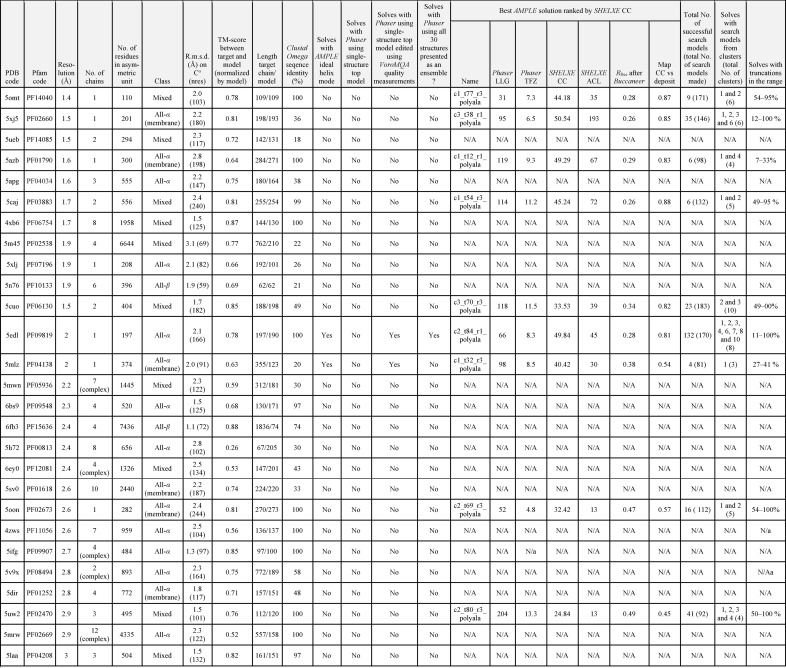

**Table 2:** Results for the seven test cases that were trialled in *AMPLE* by Rosetta remodelling of PconsFam models