# Supplementary material for: Molecular replacement using structure predictions from databases
Source: Acta Crystallogr D Struct Biol. 2019 Nov 19;75(Pt 12):1051–62. doi: 10.1107/S2059798319013962 (PMC6889911; doi:10.1107/S2059798319013962)
Supplement: Supplementary file 1 [file d-75-01051-sup1.pdf]

STRUCTURAL
BIOLOGY

Volume 75 (2019)

Supporting information for article:

Molecular replacement using structure predictions from databases

Adam J. Simpkin, Jens M. H. Thomas, Felix Simkovic, Ronan M. Keegan and Daniel J. Rigden

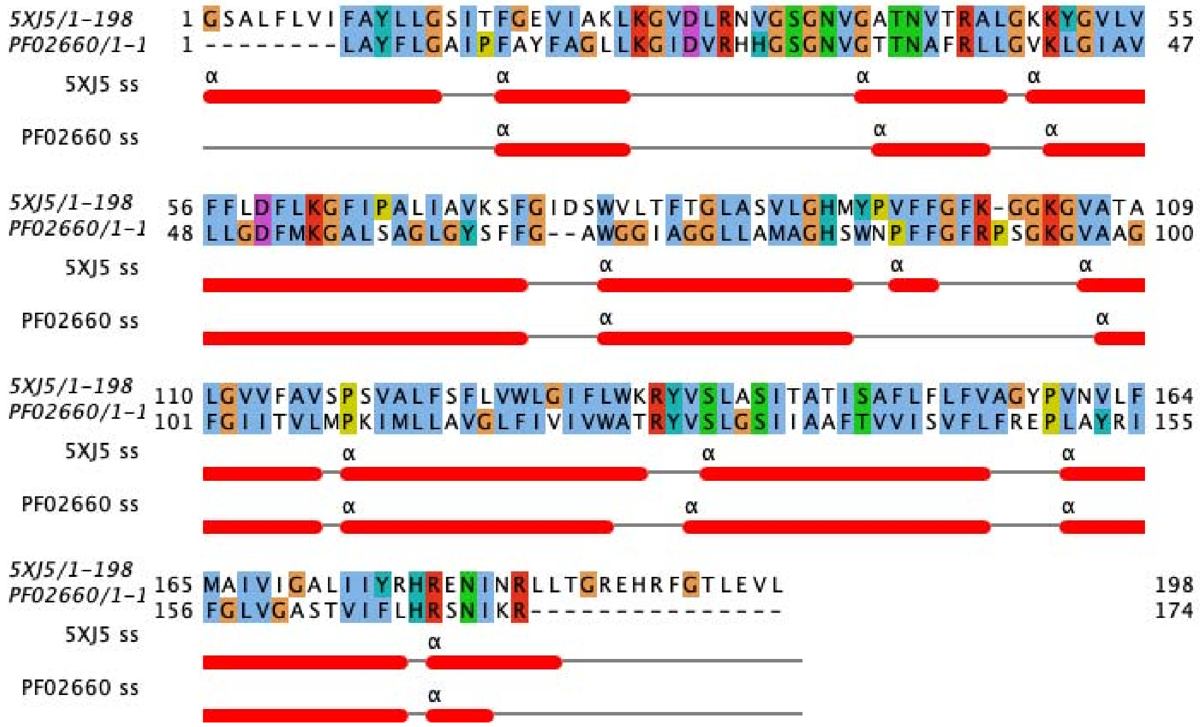

Figure S1 Sequence alignment between 5XJ5 and the model for Pfam code: PF02660. The alignment is coloured using the Clustal x colour scheme (Thompson *et al.*, 2002), and the predicted secondary structure predicted using DSSP (Kabsch & Sander, 1983) is shown below. Figure made using Jalview (Waterhouse *et al.*, 2009) as were Supp Figs 2 and 3.

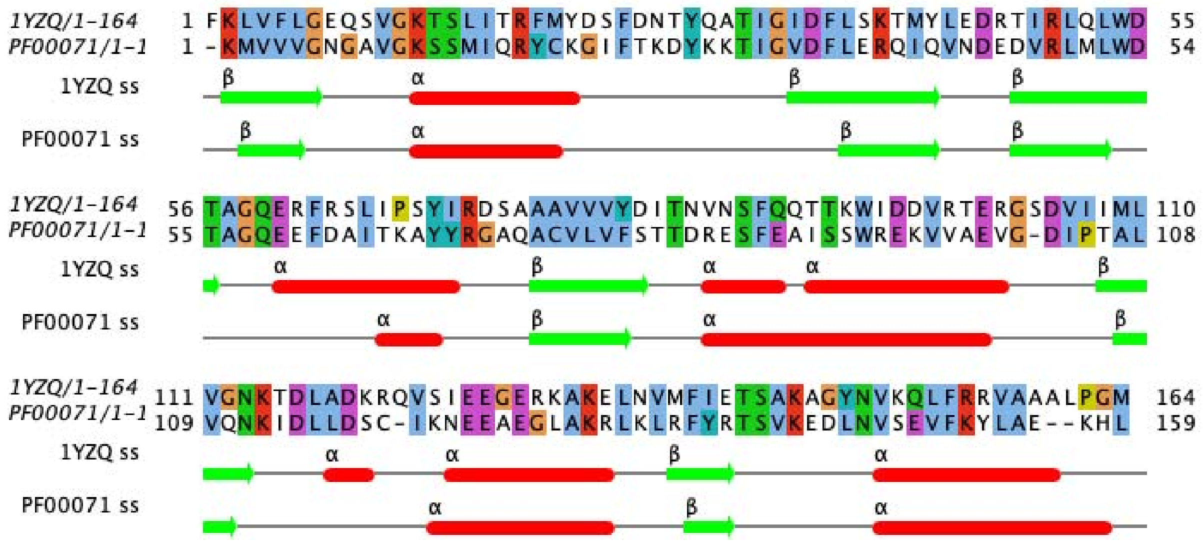

Figure S2 Sequence alignment between 1YZQ and the model for Pfam code: PF00071. The alignment is coloured using the Clustal x colour scheme, and the predicted secondary structure predicted using DSSP is shown below.

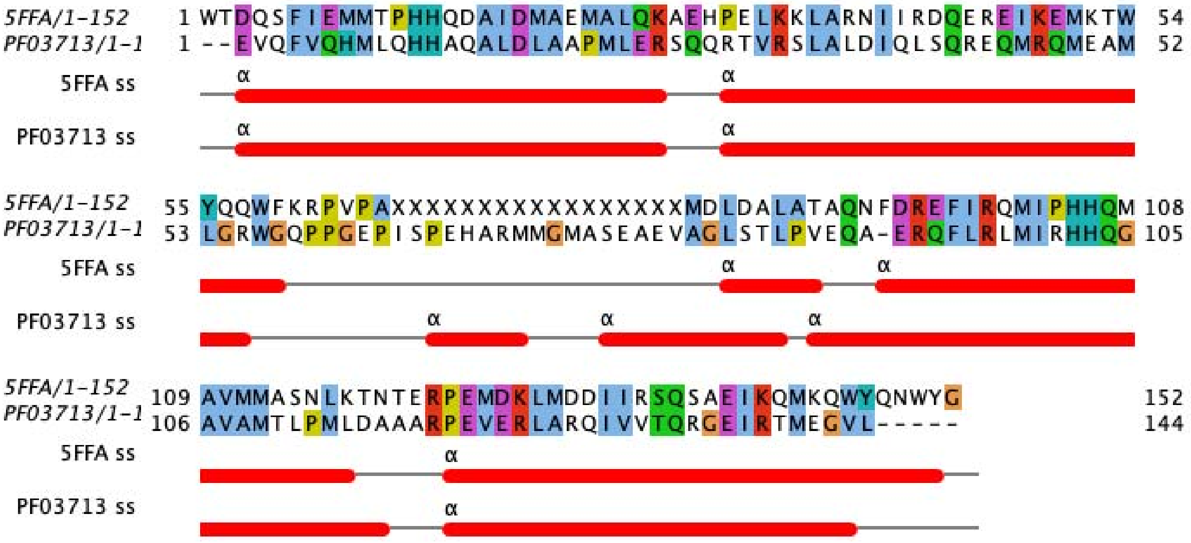

Figure S3 Sequence alignment between 5FFA and the model for Pfam code: PF03713. The alignment is coloured using the Clustal x colour scheme, and the predicted secondary structure predicted using DSSP is shown below. The X's in this alignment represent an unmodelled gap in 5FFA.

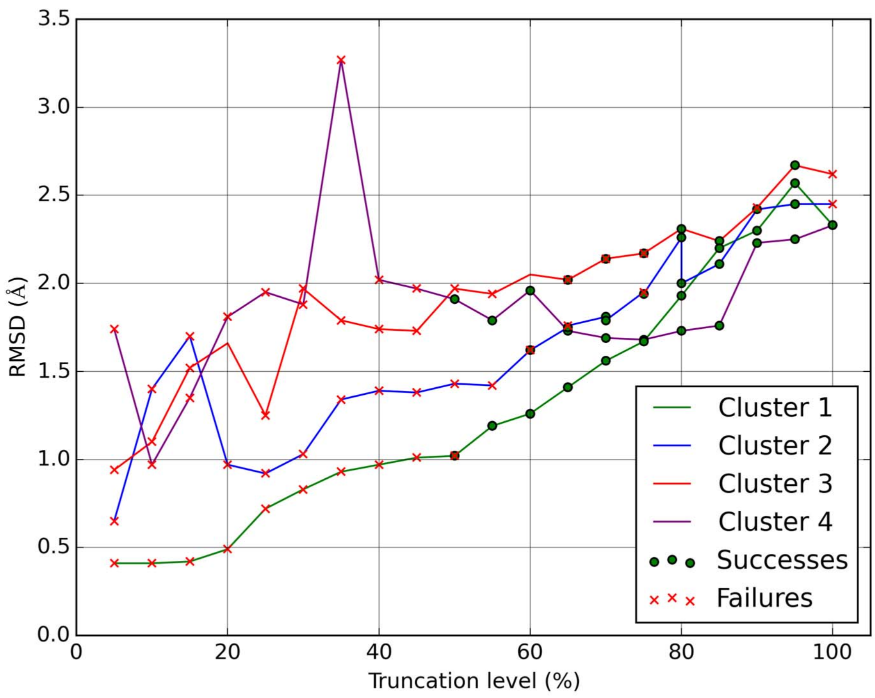

Figure S4 A comparison between the Cα RMSD (Å) calculated using TM-align (Zhang & Skolnick, 2005) and truncation level (%) for the four clusters (1, 2, 3 & 4) that contained successful search models for 5uw2. Successful models are denoted by green circles and unsuccessful models by red crosses.

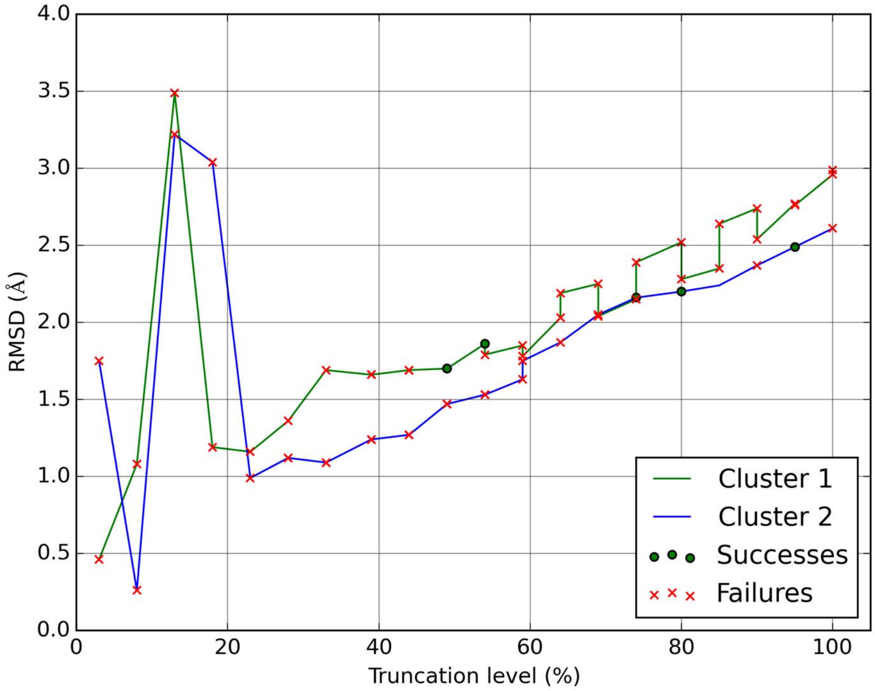

Figure S5 A comparison between the Cα RMSD (Å) calculated using TM-align and truncation level (%) for the two clusters (1 & 2) that contained successful search models for 5caj. Successful models are denoted by green circles and unsuccessful models by red crosses.

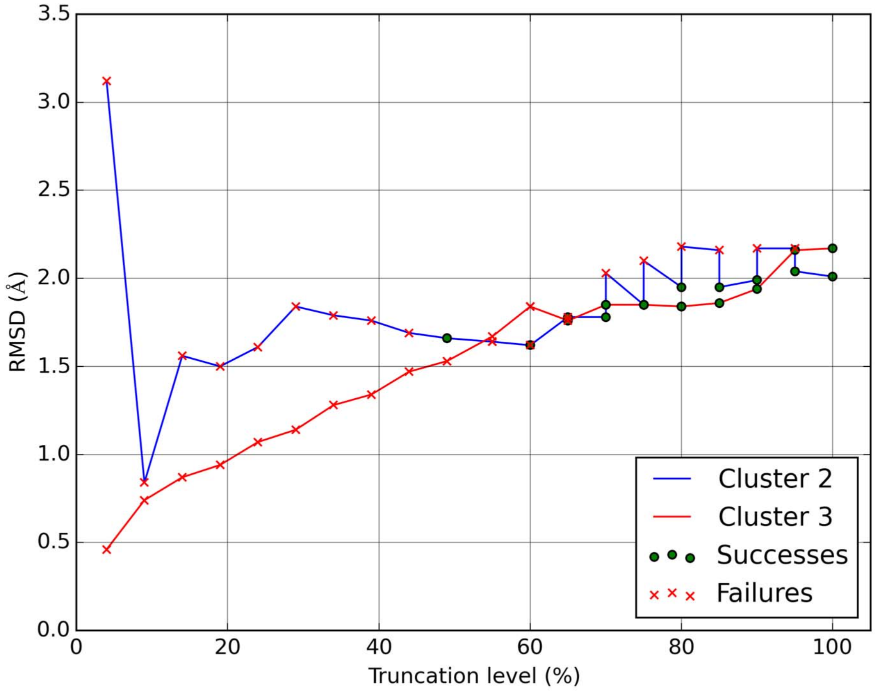

Figure S6 A comparison between the Cα RMSD (Å) calculated using TM-align and truncation level (%) for the two clusters (2 & 3) that contained successful search models for 5cuo. Successful models are denoted by green circles and unsuccessful models by red crosses.

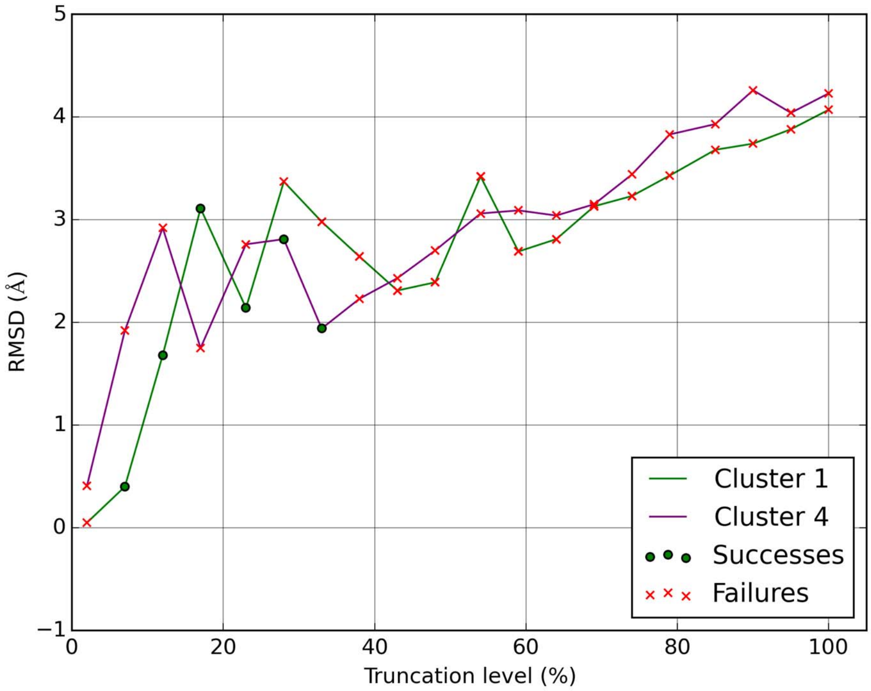

Figure S7 A comparison between the Cα RMSD (Å) calculated using TMalign and truncation level (%) for the two clusters (1 & 4) that contained successful search models for 5azb. Successful models are denoted by green circles and unsuccessful models by red crosses.

Table S1 Full table of target and model properties, and AMPLE results for the GREMLIN and PconsFam models. Rows in italics are targets not attempted due to poor resolution (>3.0A) and/or poor model quality (TM-score < 0.5) [see Excel file]

Table S2 Results of MR attempts with PconsFam models processed with CONCOORD

**Target and model properties**								**AMPLE solutions**										
pdb code	Pfam	Resolution (Å)	Number of chains	rmsd (Å) on calpha (nres)	length target chain/model	CLUSTAL Omega seqence identity	solves with AMPLE's ideal helix mode	solves using AMPLE single structure mode and VoroMQA values to edit the structure?	Map CC vs deposit	Total number of successful search models (total number of search models made)	Phaser TFZ	shelxe CC	shelxe ACL	R-free after buccaneer	Map CC vs deposit	Total number of successful search models (total number of search models made)	Solves with clusters (total number of clusters)	Solves with truncations in the range
5XJ5	PF02660	1.5	1	2.2 (162)	198/175	39	no	no	none	N/a	N/a	N/a	N/a	N/a	N/a	N/a	N/a	N/a
5AZB	PF01790	1.6	1	2.8 (193)	284/245	100	yes	no	none	N/a	N/a	N/a	N/a	N/a	N/a	N/a	N/a	N/a
1YZQ	PF00071	1.8	1	1.8 (155)	164/159	39	no	no	c8_t60_r3_polyala	42	8.3	42	53	0.33	0.292, 0.209	7 (400)	5, 6, 8, 9 (10)	50-75%
5FFA	PF03713	1.5	1	2.1 (125)	135/144	27	no	no	none	N/a	N/a	N/a	N/a	N/a	N/a	N/a	N/a	N/a

Table S3 Stereochemical quality of the PconsFam models and Rosetta derivatives

Pfam family	PDB code of crystal structure	Model source	TM-score of model vs crystal structure. Range (mean)	% Ramachandran core. Range (mean)	% Ramachandran outliers. Range (mean)	PROCHECK G-factor on dihedrals. Range (mean)
PF01790	5azb	PconsFam	0.61	74.4	6	-0.24
		Rosetta remodelling	0.25-0.60 (0.54)	75.9-87.9 (81.6)	1.0-5.0 (2.7)	-0.06-0.19 (0.05)
PF02660	5xj5	PconsFam	0.72	88.7	3.5	-0.03
		Rosetta remodelling	0.23-0.71 (0.51)	86.7-96 (91.4)	0-2.7 (0.7)	0.10-0.35 (0.23)
PF00071	1yzq	PconsFam	0.85	77.6	7.5	-0.14
		Rosetta remodelling	0.72-0.86 (0.81)	73.7-92.3 (80.9)	0-5.8 (2.7)	-0.01 – 1.14 (0.11)
PF03713	5ffa	PconsFam	0.76	95.2	1.6	0.33
		Rosetta remodelling	0.75-0.88 (0.82)	92.8-100 (96.33)	0.9-2.2 (0.1)	0.36-0.54 (0.44)

Table S4 Rotation scores derived from SIMBAD for the GREMLIN models processed by AMPLE [see Excel file]

Kabsch, W. & Sander, C. (1983). *Biopolymers.* **22,** 2577-2637.
Thompson, J. D., Gibson, T. J. & Higgins, D. G. (2002). *Curr. Protoc. Bioinformatics.* **Chapter 2,** Unit 2.3.
Waterhouse, A. M., Procter, J. B., Martin, D. M., Clamp, M. & Barton, G. J. (2009). *Bioinformatics.* **25,** 1189-1191.
Zhang, Y. & Skolnick, J. (2005). *Nucleic Acids Res.* **33,** 2302-2309.
